# An Open-Resonator Sensor for Measuring the Dielectric Properties of Antarctic Ice

**DOI:** 10.3390/s19092099

**Published:** 2019-05-07

**Authors:** Roberto Olmi, Saverio Priori, Alberto Toccafondi, Federico Puggelli

**Affiliations:** 1 IFAC-CNR, 50019 Sesto Fiorentino, Italy; s.priori@ifac.cnr.it; 2 DIISM-UNISI, 53100 Siena, Italy; albertot@dii.unisi.it (A.T.); fede.puggelli@gmail.com (F.P.)

**Keywords:** complex permittivity measurement, dielectric spectroscopy, dielectric measurement, Antarctic firn

## Abstract

In this paper, the theory behind the design of a microwave sensor for the accurate measurement of firn complex permittivity is presented. This class of microwave sensors, based on the open-coaxial re-entrant cavity method, is specifically designed to measure, by means of a simple and quick procedure, the complex permittivity profile of low loss materials. A calibration procedure is introduced to derive the complex permittivity of the material under measurement (MUM). Two specimens of this class of microwave sensors have been realized to sample the complex permittivity profile of a 106-m long ice core drilled from the Antarctic plateau at Concordia Station. The preliminary results of the on site measurement campaign are reported, showing very good agreement with theoretical models available in the literature.

## 1. Introduction

It is currently ascertained that Antarctic Peninsula climate changes, such as warming, are more rapid than in any other region on Earth [1]. Even if the causes of these changes have not yet been fully understood and probably will be the subject of scientific inquiry for a long time, it is undoubted that to proceed in understanding the mechanisms that affect the global climate changes of the Earth, we need to observe and monitor the entire geographic extent of the polar regions, including Antarctica. One of the most common methods to assess the impact of climate change is to monitor the Antarctica Ice Sheet change in mass over time. Due to Antarctica’s extent, extreme weather conditions, and remoteness, satellite remote sensing constitutes a unique tool that may provide the consistent spatial and temporal coverage necessary to estimate the ice-sheet mass balance and document the real climate and environmental changes throughout this continent [2,3,4]. The availability of new low-frequency microwave spaceborne sensors pushed the investigations further, into the deeper layers of the ice sheet and firn, down to the bedrock. However, to maximize the scientific return of these new satellite missions, corresponding in situ measurements of the firn electromagnetic characteristics, such as the complex permittivity, are required in order to calibrate and validate satellite remote sensing data. A model of the firn permittivity in the 400 MHz–2 GHz frequency is fundamental to describe the interactions of electromagnetic waves with matter, their propagation velocity, their reflections by the various layers, and the maximum depth they can penetrate, at a given frequency, into the medium. Up to now, all of the activities done to characterize the permittivity of the Antarctic cover were focused on frequencies below 250 MHz, very often up to 50 MHz [5,6]. So far, the most reliable models for the permittivity of firn in the GHz domain can predict quite well its real part (which governs wave reflections), but very poorly its imaginary part, which is responsible for wave absorption. Furthermore, the dependence of the firn permittivity on the temperature and chemical composition of the medium is not fully understood [7]. In the framework of the Italian Antarctic Program, a project has been funded with the general objective to characterize the real and imaginary parts of the firn permittivity versus depth in the 400 MHz–2 GHz microwave range by means of in situ measurements at Concordia Station. The technique mainly consists of drilling a borehole in the Antarctic firn and then measuring the real and imaginary parts of the extracted core every 5–10 cm using a dedicated sensor, followed by additional chemical analysis on each core slice. As a consequence, taking also into account the harsh measurement environments, the main requirements of the instrumentation were:

1. performing a non-destructive and non-contaminating measure;
2. allowing a fast, easy, and reliable measurement procedure;
3. providing a high sensitivity to the expected very low loss tangent of the firn permittivity.

The measurement of very low values of the loss tangent is not possible by means of reflection methods (such as the “classic” open coaxial method). Indeed, these are well known to be unsuitable for measuring both the real and the imaginary part of permittivity when these two parameters are very unbalanced [8].

Therefore, in this paper, a dedicated microwave sensor is presented that has been designed, realized, and tested for the accurate measurement of the firn complex permittivity. This is based on the open-coaxial re-entrant cavity method [9,10] typically used for the electromagnetic characterization of low loss materials. The microwave sensor was obtained by short-circuiting a coaxial section at one end, leaving the other end open to load the cavity through a firm contact with the material under measurement. The obtained cavity was loosely coupled with external circuitry by means of two ports through which a measurement of the complex transmission coefficient $S_{21}$ was performed. It was found that the transmission coefficient was vanishing except near the resonance frequencies of the cavity. Two microwave sensors have been finally designed and realized to sample the complex permittivity in the frequency range of interest (0.4–2 GHz). The first one had been designed to exhibit frequency resonances in clean air at $f_{1}=395$ MHz and $f_{2}=1.174$ GHz, while the second one exhibited frequency resonances in clean air at $f_{1}=895$ MHz and $f_{2}=2.68$ GHz. The complex permittivity of the material under measurement (MUM) was derived by comparing the measured resonance frequencies and quality factors of the loaded cavity, with that obtained by the unloaded cavity (the open end facing the air) and by loading it with a low loss dielectric material of known electrical characteristics. Tests performed on four ice samples in a cold lab ($-20{\;}^{\circ }C$) and on known samples have shown that the sensor is able to measure very low loss tangents, on the order of $\tan\delta =10^{-4}$. The paper is organized as follows. In Section 2, a circuit model of the cavity is introduced and analyzed in order to derive the relationship between the transmission coefficient and the complex permittivity of the material to be measured. In Section 3, a measurement procedure that allows deriving the unknown complex permittivity as a function of the measured parameters is presented and discussed. Finally, in Section 4, the measurement procedure is applied to derive the complex permittivity of real ice samples, and the results are reported and discussed.

## 2. The Cavity Sensor

**Figure 1:**
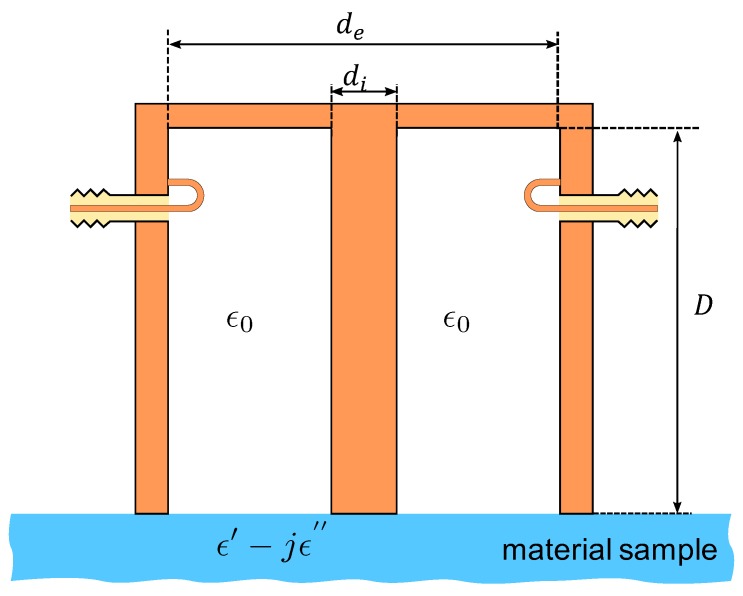
Geometry of the cavity sensor.

**Figure 2:**
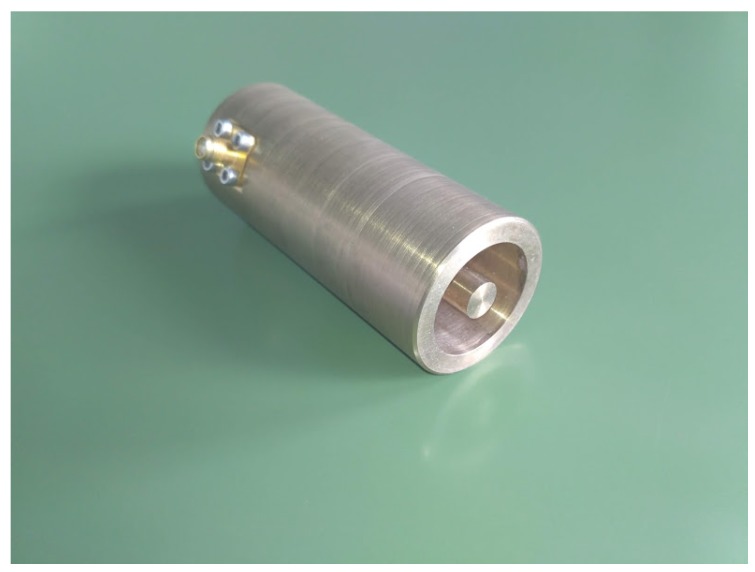
The cavity sensor operating in the higher frequency band.

Figure 1 shows the geometry of the cavity sensor. The coaxial line constituting the cavity was short circuited at one end, while the other end was open and used to probe the MUM. Figure 2 shows the fabricated cavity operating at 895 MHz and 2.68 GHz in air.

As the sensor diameter was small compared to the wavelength of the exciting signal, its radiation resistance was negligible, i.e., the sensor worked in the evanescent field region. The cavity opening electrically consisted of a capacitor-dominated impedance, the capacitance being directly related to the dielectric constant of the MUM facing the opening. In a first, very rough, approximation, a cavity of length *D* has infinite resonant frequencies, the lowest approximately corresponding to a wavelength λ equal to 4D, i.e., the cavity resonates at a frequency such that the cavity length is D=λ/4. At that frequency, the resonance mode is well known to be transverse electro-magnetic (TEM). Due to the small, but non-zero, terminating capacitance, the actual resonance frequency was slightly lower than that value or, in other words, the end capacitance made the cavity a little “electrically” longer. Higher frequency TEM resonance modes occurred at D=(2N+1)λ/4 with Nintegers, while different non-TEM higher-order modes can also occur, if excited, having a magnetic field component along the cavity axis (TE modes) or an electric field component on that axis (TM modes).

**Figure 3:**
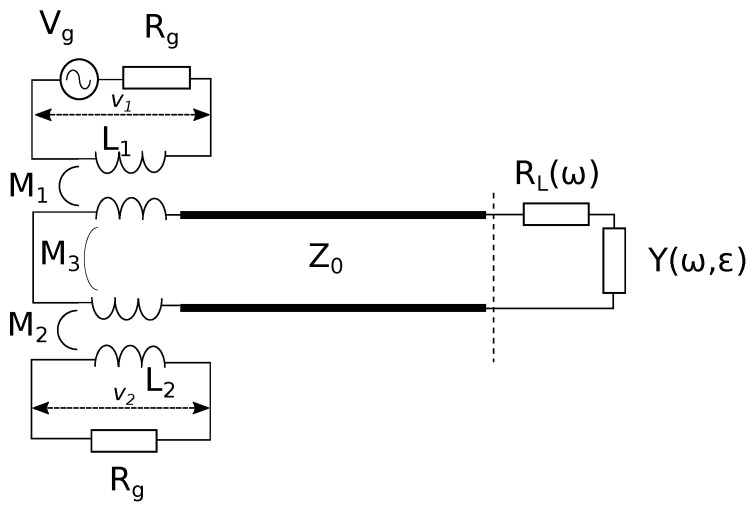
Electrical schematic of the measurement cavity sensor.

The following analysis will be limited to TEM modes, and in particular, our attention will be focused on the lowest frequency one. Figure 3 shows the equivalent circuit of the sensor. The following analysis can be easily extended to higher order modes, provided that evanescent field conditions (i.e., low radiation resistance) are fulfilled. The coaxial transmission line of characteristic impedance $Z_{0}$ constituting the cavity (thick lines in Figure 3) was connected to a vector network analyzer (VNA), represented by the generator circuit ($V_{g}$, $R_{g}$, $L_{1}$) and by the load circuit ($R_{g}$, $L_{2}$), representing the transmitting and receiving ports of the VNA. The cavity was loosely coupled to such ports, via two magnetic loops, represented by the mutual inductances $M_{1}$ and $M_{2}$. $M_{3}$ takes into account the direct coupling between the transmitting and receiving coil, whose geometrical arrangement was such that it made that parameter negligibly small. The resistance $R_{L}$ is a function of frequency, as it includes both the ohmic losses of the cavity and the generator and load resistances transformed by the mutual inductances. The coaxial line (short-circuited on the left side) terminated on an admittance Y(ω,ϵ) depending on the geometry of the coaxial aperture and on the complex permittivity of the material facing it.

The circuit can be further simplified by defining a frequency-dependent lumped inductance L(ω), which represents the impedance seen towards the short circuit from the open end of the coaxial line (dashed line in Figure 3):

(1) $$L\left(\omega \right)=\frac{Z_{0}\tan\left(\beta D\right)}{\omega }$$

where β is the wavenumber in the transmission line, corresponding to the angular frequency ω, and *D* is the cavity length.

The admittance Y(ω,ϵ) essentially consists of a complex capacitance $C\left(\epsilon \right)=C^{\prime }-jC^{\prime \prime }$ having in parallel a conductance *G* representing the radiation resistance of the coaxial opening [11]. If the coaxial size is small with respect to the wavelength, the cavity radiation is negligibly small, and the *G* term can be included in the frequency-dependent resistance $R_{L}\left(\omega \right)$. The capacitance *C* is a complicated function of the mode structure of the electric field at the coaxial opening [8,12]. However, a simple analysis can be conducted, based on the Deschamps theorem [13], establishing a relationship between the open admittance in air, Y(ω,1), and that on the material of (relative) complex permittivity $\epsilon =\epsilon^{\prime }-j\epsilon^{\prime \prime }$, Y(ω,ϵ):

(2) $$Y\left(\omega ,\epsilon \right)=\sqrt{\epsilon }Y\left(\omega \sqrt{\epsilon },1\right)$$

Equation (2) asserts that the admittance in the material is $\sqrt{\epsilon }$ times the admittance in air at the “scaled” frequency $\omega \sqrt{\epsilon }$.

Denoting by $C_{a}$ the capacitance when air terminates the cavity sensor (i.e., without the MUM), the admittance corresponding to a material of complex permittivity ϵ is $Y\left(\epsilon \right)=j\omega \epsilon^{\prime }C_{a}+\omega \epsilon^{\prime \prime }C_{a}$.

The quantity measured by the VNA is the transmission coefficient between Ports 2 and 1, $S_{21}$, consisting of the ratio between the voltages denoted by $v_{2}$ and $v_{1}$ in the electrical schematic. Straightforward calculations give the following expression [10]:

(3) $$S_{21}=\left|\frac{v_{2}}{v_{1}}\right|=\left|\frac{\alpha R_{g}}{j\omega L_{1}+j\omega \alpha M_{3}+\omega^{2}\frac{M_{1}^{2}}{Z_{s}}-\alpha \omega^{2}\frac{M_{1}M_{2}}{Z_{s}}}\right|$$

with:

$$Z_{s}=R_{L}\left(\omega \right)+j\omega L\left(\omega \right)+\frac{1}{j\omega C(\epsilon )}$$

$$\alpha =\frac{\omega^{2}M_{1}M_{2}-j\omega M_{3}Z_{s}}{\omega^{2}M_{2}^{2}+\left(R_{g}+j\omega L_{2}\right)Z_{s}}$$

Note that the inductances in the “secondary” circuit including the cavity have been implicitly included in L(ω). It can be easily shown that the frequency behavior of $S_{21}$, at least in the region around the resonance peak, can be well described by an expression like:

(4) $$S_{21}=\frac{A\omega }{\left|Z_{s}\left(\omega \right)\right|}$$

where *A* is a constant. In other words, the information about the real and imaginary part of the MUM permittivity (which are related to the resonance frequency and quality factor) is contained in the zeros of the series impedance $Z_{s}$ (the dependence on ω has been explicitly indicated in (4)); hence, an analysis of the series impedance of the cavity is all we need.

## 3. Measurement Procedure

The measurement of the dielectric properties of an unknown material ($\epsilon_{m}$) requires determining the unknown parameters in $Z_{s}$. To accomplish this, we performed two “calibration” measurements, with the cavity facing air and with the cavity placed on a dielectric material with known dielectric permittivity. In this case, we used the Eccostock${}^{®}$0005 (ES5), a low loss, cross-linked, polystyrene having certified dielectric properties (flat on a very large frequency band) that were: $\epsilon^{\prime }=2.53$ and $\tan\delta =5\times 10^{-4}$.

### 3.1. Calibration Facing Air

**Figure 4:**
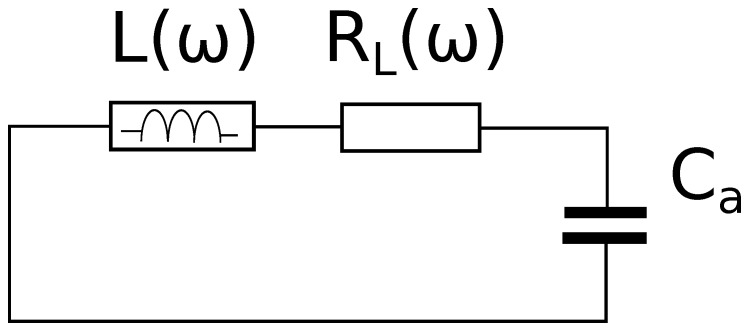
Equivalent circuit of the cavity facing air.

In Figure 4 is shown the series impedance of the resonant cavity equivalent circuit when the open-end was facing the free-space.

The resonance condition corresponds to $Im(Z_{s})=0$. Of course, this happens at an angular frequency $\omega_{a}$ such that:

(5) $$\omega_{a}L\left(\omega_{a}\right)\equiv Z_{0}\tan\left(\beta_{a}D\right)=\frac{1}{\omega_{a}C_{a}}$$

Equation (5) allows obtaining the cavity termination capacitance in air $C_{a}$.

The quality factor is defined as usual:

(6) $$Q_{a}=\frac{Z_{0}\tan\left(\beta_{a}D\right)}{R_{L}\left(\omega_{a}\right)}$$

### 3.2. Calibration Facing ES5

**Figure 5:**
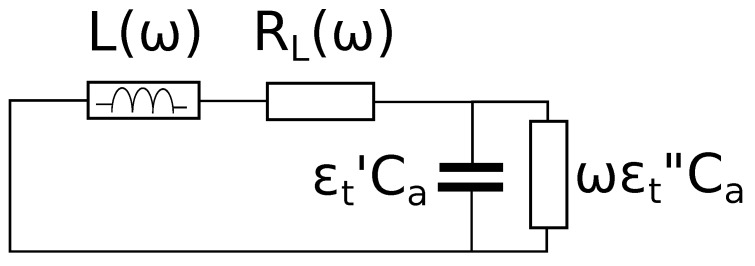
Equivalent circuit of the cavity facing the calibration material ES5.

In Figure 5 is shown the equivalent circuit for the resonant cavity when the open-end was facing the ES5 dielectric material.

From Equation (2), we obtain the expression of the termination admittance when the cavity opens onto the “test” material ES5:

(7) $$Y_{t}\left(\omega ,\epsilon_{t}\right)=\sqrt{\epsilon_{t}}Y\left(\sqrt{\epsilon_{t}}\omega ,1\right)=j\omega \epsilon_{t}C_{a}$$

where $\epsilon_{t}=\epsilon_{t}^{\prime }-j\epsilon_{t}^{\prime \prime }$ is the complex permittivity of ES5.

The “full” termination admittance, when the cavity open-end faces a dielectric of complex permittivity $\epsilon_{t}$, should be given by the parallel between the termination admittance (7) and the radiation conductance of the aperture. This last is a function of the frequency and should be conveniently included in the total frequency-dependent resistance of the cavity. However, as long as the radiation conductance of the aperture can be neglected, the termination susceptance is practically given by:

$$B_{t}=\omega \epsilon_{t}^{\prime }C_{a}$$

while the termination conductance is:

$$G_{t}=\omega \epsilon_{t}^{\prime \prime }C_{a}$$

Denoting by $R_{a}$ the series resistance of the admittance terminating the line in Figure 5, the quality factor is now:

(8) $$Q_{t}=\frac{Z_{0}\tan\left(\beta_{t}D\right)}{R_{L}\left(\omega_{t}\right)+R_{a}}$$

where $\beta_{t}$ is the propagation constant and $\omega_{t}$ the resonance angular frequency, the subscript “*t*” referring to the resonance conditions with the “test” material terminating the cavity, and:

(9) $$R_{a}=\frac{\epsilon_{t}^{\prime \prime }}{\omega_{t}C_{a}{\left|\epsilon_{t}\right|}^{2}}$$

In (8), $R_{L}$ is not known at the angular frequency $\omega_{t}$, because using a single cavity, we are not able to estimate the frequency behavior of $R_{L}$. To obtain the ω dependence of $R_{L}$, a numerical model has been implemented, as described in the following subsection.

### 3.3. Frequency Dependence of the Total Cavity Wall Resistance

**Figure 6:**
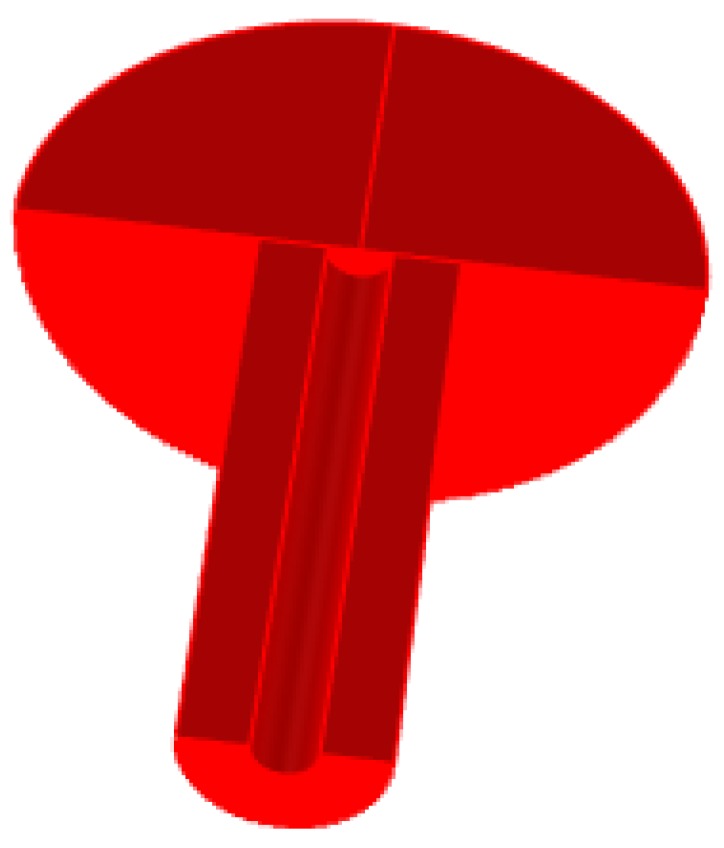
The axially-symmetrical model of the cavity sensor.

A finite-element (FE) model of the cavity sensor has been implemented using COMSOL Multiphysics^®^. The model only represents the “secondary” circuit (shown in Figure 4), and it is axially symmetric. Figure 6 shows a cross-section of the model: the semi-spherical region closing the cavity on the “open” side has dimensions such that it negligibly perturbs the electric field distribution at the cavity aperture.

The electrical conductivity assigned to the cavity walls was such that it gave the experimentally-measured $Q_{a}$ at the resonance angular frequency $\omega_{a}$. Such an effective conductivity takes into account all loss sources, e.g., the actual ohmic losses of the walls and the losses transported into the secondary circuit by the two primary circuits (generator and load) shown in Figure 3. The semi-spherical region was assumed as a perfect-electric conductor.

In order to compute the frequency dependence of $R_{L}$, we have to change the resonant frequency of the cavity without changing its geometrical length. The only way to do this is to change the dielectric constant of the material filling the semi-spherical region, of course without introducing spurious losses (i.e., assuming purely real permittivity values). For every dielectric constant value, the FE model allows computing a complex resonance frequency $\tilde{\omega_{r}}=\omega_{r}\left(1+\frac{j}{2Q_{r}}\right)$, where $\omega_{r}=Re\left(\tilde{\omega_{r}}\right)$, and therefore:

$$Q_{r}=\frac{\omega_{r}}{2Im(\tilde{\omega_{r}})}$$

**Figure 7:**
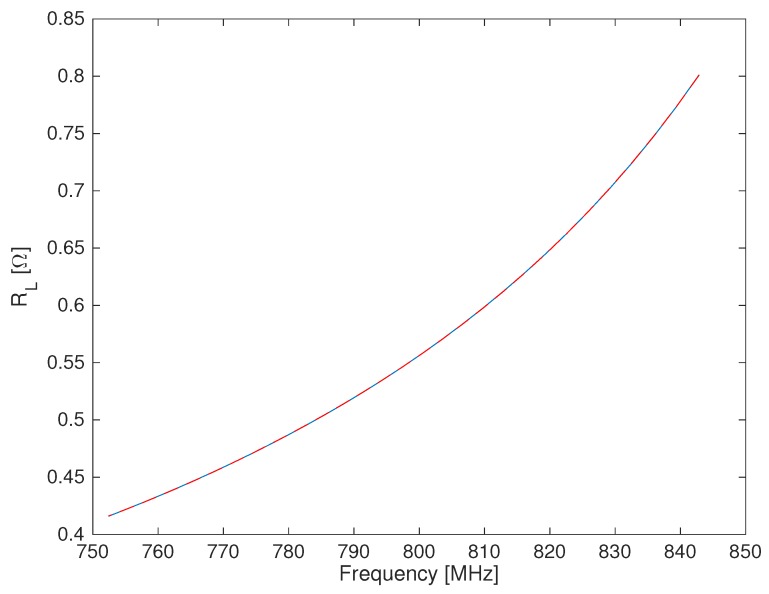
Computed frequency-dependent total resistance of the cavity sensor walls.

As a last step, $R_{L}$ is computed at each frequency by means of (6). Figure 7 shows the calculated frequency dependence of $R_{L}$. The frequency dependence of the resistance is a fourth-order polynomial. Such a behavior is very similar to what has been experimentally determined for re-entrant cavity devices [10], where changing the termination capacitance in air is possible. At this point, a doubt could arise about the possibility of avoiding the numerical model and experimentally measuring $R_{L}\left(\omega \right)$. As we noted above, to change the resonance frequency, we have to change the termination capacitance. To do that experimentally, without introducing additional losses, we must have available a certain number of loss-free dielectrics, but of course, we have not. An alternative could be to use real dielectrics (i.e., with losses, however small they are) with precisely-known loss tangent values and to take into account those loss values. That is feasible, in principle, but not very easy to realize.

### 3.4. Measurement on Unknown Material

**Figure 8:**
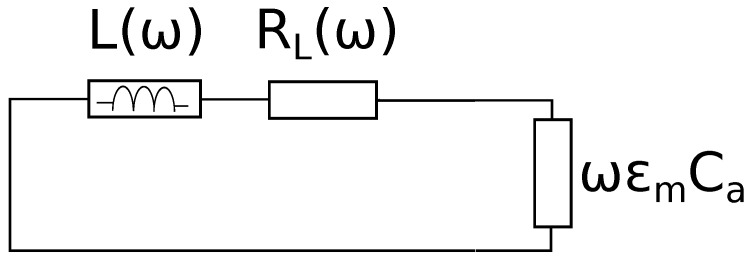
Equivalent circuit of the cavity facing the material under measurement (MUM).

The equivalent circuit of the measurement is represented in Figure 8. The unknown dielectric constant $\epsilon_{m}^{\prime }$ and loss tangent $\tan\delta_{m}$ were computed from the measured resonance frequency $\omega_{m}$ and quality factor $Q_{m}$, respectively. The subscript “*m*” refers to the material under measurement.

The dielectric constant comes from equating to zero the total impedance of the resonant circuit. This latter is the series among the impedances at the left and at right of the capacitor:

(10) $$Z_{s}=Z_{0}\left(\omega_{m}\right)\tanh\left(\gamma_{m}D\right)-j\frac{1}{\omega_{m}\epsilon_{m}C_{a}}$$

where the propagation constant $\gamma_{m}$ and the characteristic impedance $Z_{0}\left(\omega_{m}\right)$ are complex quantities because the coaxial line constituting the cavity is a (low) lossy line.

Expressing the $C_{a}$ air capacity in terms of the measured resonance frequency in air (5), we obtain:

(11) $$\epsilon_{m}=\frac{jZ_{0}\left(\omega_{a}\right)\omega_{a}\tan\left(\beta_{a}D\right)}{Z_{0}\left(\omega_{m}\right)\omega_{m}\tanh\left(\gamma_{m}D\right)}$$

It is easy to show that for low loss materials, and for a very low loss coaxial line (as needed for having a high Qvalue), the characteristic impedance $Z_{0}$ is practically real and frequency independent and (11) becomes:

(12) $$\epsilon_{m}^{\prime }=\frac{\omega_{a}\tan\left(\beta_{a}D\right)}{\omega_{m}\tan\left(\beta_{m}D\right)}$$

where $\beta_{m}=Im\left(\gamma_{m}\right)$

The loss tangent $\tan\delta_{m}$ is computed from (8) and (9). If $\epsilon_{m}^{\prime \prime }\ll \epsilon_{m}^{\prime }$, we obtain:

(13) $$\tan\delta_{m}=\omega_{m}\epsilon_{m}^{\prime }C_{a}\left[\frac{Z_{0}\tan\left(\beta_{m}D\right)}{Q_{m}}-R_{L}\left(\omega_{m}\right)\right]$$

Actually, Expressions (12) and (13), which only depend on the measurement on the unknown material and on air, must usually be corrected taking into account the measurement on a material of known properties (close to those of the material of interest), in our case the ES5 (see Section 3.2). For the purpose of obtaining a higher precision, for example, Expression (12) must take into account the presence of a very small thickness of air between the sensor head and part of the specimen, which results in a series capacitance $C_{s}$ given in terms of the $\epsilon_{t}^{\prime }$ computed by (12) and the “true” value $\epsilon_{tc}^{\prime }$:

(14) $$C_{s}=\frac{\epsilon_{tc}^{\prime }C_{a}}{\epsilon_{tc}^{\prime }/\epsilon_{t}^{\prime }-1}$$

As a consequence, assuming the same average air thickness for the unknown material (the flatness of the specimen surface is a prerequisite; therefore, the presence of air is mainly due to small imperfections in the sensor head), the corrected $\epsilon_{mc}^{\prime }$ is obtained from (12) by:

(15) $$\epsilon_{mc}^{\prime }=\frac{\epsilon_{m}^{\prime }C_{s}}{C_{s}-\epsilon_{m}^{\prime }C_{a}}$$

The loss tangent given by (13) is corrected multiplying it by a correction factor involving the quality factor measured with ES5 ($Q_{t}$) and the quality factor in air ($Q_{at}$) “translated” to the same resonant frequency $\omega_{t}$:

(16) $$\kappa_{t}=\frac{\tan\delta_{tc}}{1/Q_{t}-1/Q_{at}}$$

where $\tan\delta_{tc}$ is the “true” loss tangent value of ES5. Eventually, the loss tangent value of the material is given by:

(17) $$\tan\delta_{mc}=\kappa_{t}\tan\delta_{m}$$

### 3.5. Sensing Depth

The sensitivity of the sensor resonance frequency ($f_{r}$) and quality factor (*Q*) on the sample thickness have been investigated numerically. The FEM simulation computed the complex frequency of the first eigenmode of the resonant cavity terminated by an ice sample backed by a metallic plate. To investigate the worst-case conditions, ice was assumed to have the permittivity of superficial firn ($\epsilon^{\prime }=1.5$, $\tan\delta =5\times 10^{-4}$). A greater value of permittivity further reduces the error due to the metal perturbation. The metal termination, although not realistic (usually samples are not placed on a metallic support), is clearly the worst-possible condition for the electric field.

**Figure 9:**
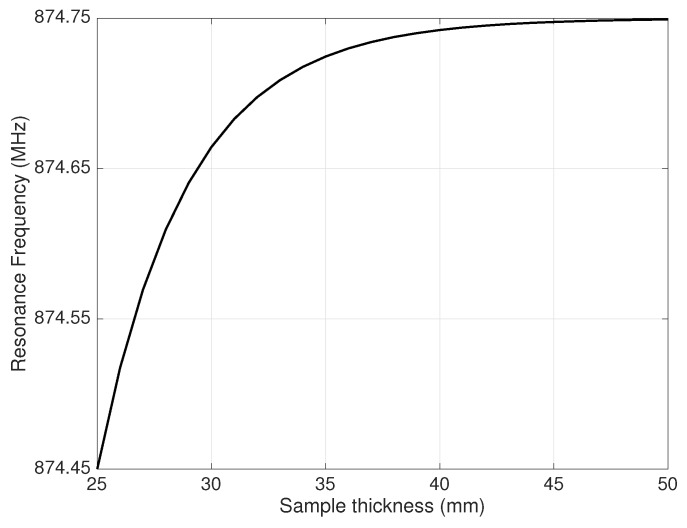
Sensitivity of resonance frequency to the sample thickness.

Figure 9 shows how the resonance frequency depends on the sample thickness. The relative percentage error on $f_{r}$ decreasing the thickness from 5–2.5 cm was about 0.03%.

**Figure 10:**
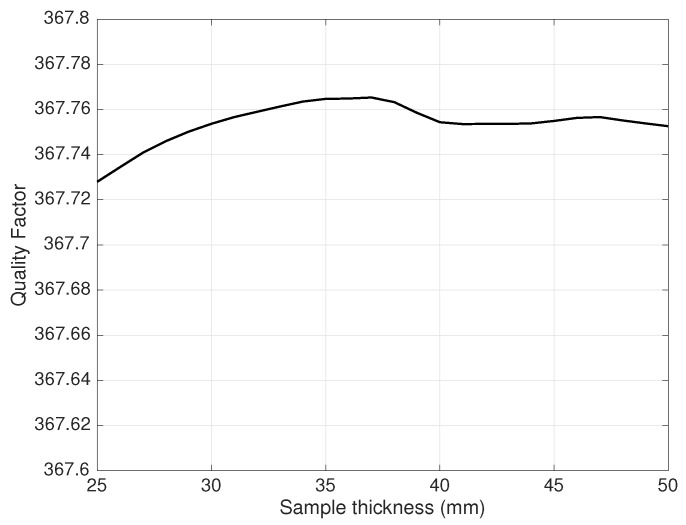
Sensitivity of *Q* to the sample thickness.

Analogously, Figure 10 shows how the resonant cavity loaded *Q* depends on the sample thickness. The relative percentage variation on *Q* on the thickness range 2.5–5 cm was about 0.01%.

As the dielectric constant $\epsilon^{\prime }$ is mainly (and almost linearly) related to $f_{r}$, while the loss tangent tanδ is mainly related to *Q*, we expect similar errors on the dielectric parameters, due to the finiteness of the sample thickness.

The correctness of the above analysis has been verified experimentally by measurements on Teflon samples of different thickness backed by a metallic plate. The dielectric parameters obtained did not depend on the thickness, if the samples were thicker than a couple of cm.

## 4. Results and Discussion

The measurement procedure has been initially tested on Teflon^®^, which is a commercial name for polytetrafluoroethylene, since its dielectric properties slightly change among manufacturers. From data found in the literature (e.g., [14,15]), Teflon’s dielectric constant is between 2.0 an 2.1 in a wide frequency range (up to several GHz), and its loss tangent is usually between $1\times 10^{-4}$ and $4\times 10^{-4}$. Using the first of the two fabricated sensors, the measured value at the frequency resonance of 860 MHz was: $\epsilon_{Tef}=2.03$, $\tan\delta_{Tef}=1.4\times 10^{-4}$. These results are in a very good agreement with those typically found in literature.

In preparation for the Antarctic measurement campaign, four ice samples have been prepared and measured in a cold lab at $-20\;{}^{\circ }C$. One of the samples was obtained from de-ionized water, and the other three from saline solutions having NaCl concentrations of 0.1, 0.01, and 0.001 moles/liter. Table 1 shows the measurement results at the reference frequency resonance of 840 MHz.

The results on Teflon and those reported in Table 1 showed that the measurement method was able to measure very low loss tangents. On the other hand, while the dielectric constant of the teflon sample was properly obtained, the values for ice suffered for a systematic error, as the correct value of the ice permittivity is known to be close to 3.15. The low dielectric constant values for low-salinity ice (first three rows of the table) were probably due to an imperfect contact between the probe and the ice surface or, in other words, to the presence of air between the probe and the material. The large error in the dielectric constant of the high-salinity ice was due to the material consistency, which, due to the massive presence of salt, was like a gel rather than a hard solid.

Although the relative error on ϵ was not so huge in the first three cases (the measured permittivity was about 14% lower than the expected one), we were warned to obtain an as smooth as possible surface of the samples during the Antarctic campaign.

### 4.1. Preliminary Results on Antarctic Firn

**Figure 11:**
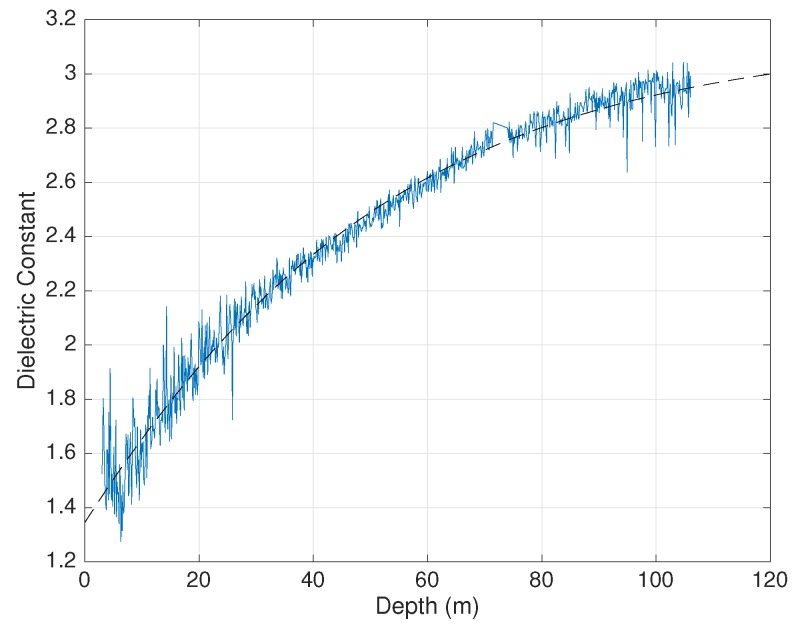
Measured dielectric constant at about 850 MHz, as a function of depth.

This section reports preliminary results of measurements conducted in an ice/firn carrot drilled out from the Antarctic pack close to Concordia Station on December 2018. The carrot, about 106 m long, has been cut in slices of thicknesses around 10 cm. Figure 11 shows the measured dielectric constant as a function of depth (blue line). The measurement frequency varied between 830 MHz (deeper samples) and 880 MHz (superficial samples). The first usable samples were at a depth of about 3 m, because more superficial samples deteriorated during the drilling/extraction phase.

It is well known that the dielectric constant $\epsilon^{\prime }$ is related to the ice specific density ρ (ice density relative to that of liquid water at $4{\;}^{\circ }C$) by the following expression [6]:

$$\epsilon^{\prime }={\left(1+\gamma \rho \right)}^{2}$$

where γ=0.845. Moreover, the specific density of ice is an exponential function of the depth (see, for example, [16]), such that the depth dependence of the dielectric constant is like:

(18) $$\epsilon^{\prime }\left(z\right)={\left(1.77-\alpha e^{-\beta z}\right)}^{2}$$

The fitting function (18), fulfilling the condition for pure ice:

$$\lim_{z\to \infty }\epsilon^{\prime }\approx 3.15$$

is also shown in Figure 11 (dashed line). It is worth noting that the fitting function was well superimposed on the experimental data. Incidentally, the β constant obtained from the fitting corresponded to a “decay length” 1/β of the order of 45 m, consistent with the physical situation.

**Figure 12:**
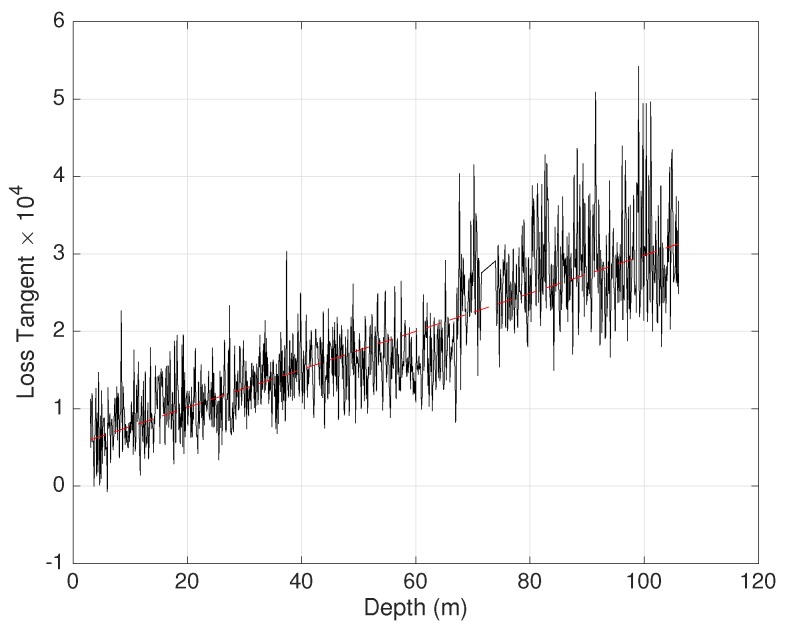
Measured loss tangent at about 850 MHz, as a function of depth.

In Figure 12, the measured loss tangent as a function of depth is shown (black line). It was found that loss tangent was rather variable with depth, varying between $0.5\times 10^{-4}$ and $5\times 10^{-4}$. However, the fitted value (red line) was monotonically growing with depth varying from $0.5\times 10^{-4}$–$3\times 10^{-4}$.

### 4.2. Conclusions

In this paper, the theory behind the design of a microwave sensor for the accurate measurement of firn complex permittivity was presented, based on the open-coaxial re-entrant cavity method. Such a microwave sensor has been specifically designed to measure, by means of a simple and quick procedure, the complex permittivity profile of low loss materials, such as firn. A calibration procedure has been introduced to derive the complex permittivity of the material under measurement (MUM). This was obtained by comparing the measured resonance frequencies and quality factors of the cavity with the MUM, with that obtained by the unloaded cavity (the open end facing clean air) and by loading it with a low loss dielectric material of known electrical characteristics. This allows one to attain a very good degree of accuracy in the estimation of the loss tangent of the MUM, as confirmed by tests on known materials. Two specimens of this class of microwave sensors have been finally realized to sample the complex permittivity profile, in the frequency range of interest (0.4–2 GHz), of a 106 m-long ice core drilled from the Antarctic plateau at Concordia Station. Preliminary results, at a frequency of 868 MHz, showed that the measured profile was in very good agreement with those calculated by theoretical models.

## Figures and Tables

**Table 1:** Measured complex permittivity of several saline solutions.

$c_{\mathrm{NaCl}}$ (moles/liter)	$\epsilon^{\prime }$	tanδ
0	2.7	$3.8\times 10^{-4}$
0.001	2.7	$5.0\times 10^{-4}$
0.01	2.7	$1.4\times 10^{-3}$
0.1	2.4	$8\times 10^{-3}$
